# Identification and experimental validation of a tumor-infiltrating lymphocytes–related long noncoding RNA signature for prognosis of clear cell renal cell carcinoma

**DOI:** 10.3389/fimmu.2022.1046790

**Published:** 2022-11-24

**Authors:** Yulin Deng, Kai Guo, Zhenfeng Tang, Yuanfa Feng, Shanghua Cai, Jianheng Ye, Yuanxue Xi, Jinchuang Li, Ren Liu, Chao Cai, Zeheng Tan, Yixun Zhang, Zhaodong Han, Guohua Zeng, Weide Zhong

**Affiliations:** ^1^ Department of Urology, Minimally Invasive Surgery Center, Guangdong Key Laboratory of Urology, Guangzhou Urology Research Institute, The First Affiliated Hospital of Guangzhou Medical University, Guangzhou Medical University, Guangzhou, Guangdong, China; ^2^ Department of Urology, Zhujiang Hospital of Southern Medical University, Guangzhou, Guangdong, China; ^3^ Guangzhou Laboratory, Guangzhou Medical University, Guangzhou, Guangdong, China; ^4^ Department of Urology, Guangdong Key Laboratory of Clinical Molecular Medicine and Diagnostics, Guangzhou First People’s Hospital, School of Medicine, South China University of Technology, Guangzhou, Guangdong, China; ^5^ State Key Laboratory of Quality Research in Chinese Medicines, Macau University of Science and Technology, Macau, Macau SAR, China

**Keywords:** tumor-infiltrating lymphocytes, long noncoding RNA, bioinformatics, clear cell renal cell carcinoma, prognostic

## Abstract

Clear cell renal cell carcinoma (ccRCC) is a common aggressive malignant tumor of the urinary system. Given the heterogeneity of the tumor microenvironment, immunotherapy may not fully exert its role in the treatment of advanced patients. Long noncoding RNA (lncRNA) has been reported to be critically associated with the differentiation and maturation of tumor-infiltrating lymphocytes (TILs), which work against tumor cells. In this study, we identified 10 TIL-related lncRNAs (AL590094.1, LINC02027, LINC00460, AC147651.1, AC026401.3, LINC00944, LINC01615, AP000439.2, AL162586.1, and AC084876.1) by Pearson correlation, univariate Cox regression, Lasso regression, and multivariate Cox regression based on The Cancer Genome Atlas (TCGA) database. A risk score model was established based on these lncRNAs. Next, a nomogram was constructed to predict the overall survival. By employing differentially expressed genes (DEGs) between groups with high and low risk scores, gene ontology (GO) enrichment analysis was performed to identify the major biological processes (BP) related to immune DEGs. We analyzed the mutation data of the groups and demonstrated that SETD2 and BAP1 had the highest mutation frequency in the high-risk group. The “CIBERSORT” R package was used to detect the abundance of TILs in the groups. The expression of lymphocyte markers was compared. We also determined the expression of two lncRNAs (AC084876.1 and AC026401.3) and their relationship with lymphocyte markers in the kidney tissue of ccRCC patients and showed that there was a positive correlation between AC084876.1 and FoxP3. Proliferation, migration, and invasion of AC084876.1-downregulated ccRCC cell lines were inhibited, and the expression of PD-L1 and TGF-β secretion decreased. To our knowledge, this is the first bioinformatics study to establish a prognostic model for ccRCC using TIL-related lncRNAs. These lncRNAs were associated with T-cell activities and may serve as biomarkers of disease prognosis.

## Introduction

Renal cell carcinoma (RCC) is a common malignant tumor of the urinary system. Globally, 431,288 new cases (accounting for 2.2% of all cancer cases) and 179,368 new deaths (accounting for 1.8% of all cancer deaths) occurred in 2020, second only to prostate cancer and bladder cancer (1). In 2021, the number of new cases of kidney cancer in the United States was 76,080, ranking sixth in the male cancer incidence rate and ninth in the female cancer incidence rate (2). RCC has several subtypes, and about 70% of individuals receive a diagnosis of clear cell RCC (ccRCC). Although ccRCC is a disease that can be detected early and successfully treated by surgery, up to one-third of cases relapse or develop metastasis (3). ccRCC is not sensitive to radiotherapy and chemotherapy. The application of immunotherapy for different targets brings hope to these patients (4, 5); however, the effective response rate of the treatment is limited. Therefore, it is necessary to find new effective immunotherapeutic targets to improve patients’ prognoses.

Immune cells in the tumor microenvironment play an important role in regulating tumor progression and serve as attractive therapeutic targets (6). Moreover, ccRCC is prone to immune infiltration, and the characteristics of the tumor microenvironment strongly affect the response to immunotherapy (7). A successful antitumor immune response requires the activation and synergy of multiple tumor-infiltrating lymphocytes, including T cells, B cells, natural killer cells (NK cells), and their subtypes. These cells play a positive and negative regulatory role in the process of antitumor immunity and kill tumor cells (8). Available studies have constructed signatures related to immune infiltration based on different biological characteristics in ccRCC. Moreover, specific lymphocyte related signatures such as CD8^+^ T cells (9), CD39^+^CD8^+^ T cells (10), and TNFRSF9^+^CD8^+^ T cells (11) have been identified in ccRCC. However, previous studies did not focus on the impact of tumor-infiltrating lymphocyte profile on the prognosis of patients with ccRCC, so they could not accurately demonstrate the potential role of immune cells in the treatment of ccRCC.

Long noncoding RNAs (lncRNA) have a length of over 200 nucleotides and are dynamically expressed in the immune system and regulate the differentiation and function of immune cells (12). Previous studies have shown that lncRNAs are dysregulated in cancer and that they have important effects on tumor proliferation, angiogenesis, apoptosis, and metastasis by regulating the formation of the tumor immune microenvironment (13). For instance, lncRNA LIMIT has been identified to be associated with tumor-infiltrating T cells. Silencing LIMIT could impair antitumor immunity and blunt immunotherapy efficacy (14). Breast cancer–derived exosomal lncRNA SNHG16/miR-16-5p/SMAD5-regulatory axis can induce CD73 expression in γδT cells, thereby enhancing the immunosuppressive function (15). LINC00301 is highly expressed in non-small cell lung cancer and functions to increase regulatory T cells while decreasing the CD8+ T cell population (16). A clinical trial suggested that lncRNA was associated with immunotherapeutic overall survival benefits superior tumor alteration burden, programmed cell death ligand 1 (PD-L1) expression, and cytotoxic T-lymphocyte (CTL) infiltration (17). Based on the above findings, lncRNAs are considered a potential target for immunotherapy and have attracted extensive attention in the field of cancer treatment research.

With the maturation in the application of high-throughput sequencing technology, it has become possible to explore the expression levels of lncRNAs in ccRCC and their relationship with tumor-infiltrating lymphocytes; additionally, bioinformatics analysis can be applied to further explore the relevant mechanisms. In this study, we identified a signature consisting of 10 TIL-related lncRNAs to predict the prognosis of patients with ccRCC and explored the potential mechanism. Human renal tissues were used to detect the expression levels of related lncRNAs and immune markers. Finally, the biological functional effect of AC084876.1 on the ccRCC cell line was examined by *in vitro* experiments.

## Materials and methods

### Ethical statement

This study was approved by the Ethics Committee of Guangzhou First People’s Hospital, School of Medicine, South China University of Technology. Informed consent forms were signed by all patients. In accordance with the ethical and legal standards, seven pairs of matched frozen samples of ccRCC and benign renal tissue adjacent to cancer from ccRCC patients were handled and made anonymous.

### Cell lines

A human ccRCC cell line 786-O was obtained from ATCC (American Type Culture Collection, Manassas, Virginia, USA). The cells were cultured at 37°C in a 5% CO_2_ incubator with RPMI-1640 (MA0215, Meilunbio, Dalian, China) medium containing 10% fetal bovine serum, streptomycin, and penicillin.

### Data and tissue processing

The RNA-sequencing data and corresponding clinical information of patients with ccRCC were downloaded from the Cancer Genome Atlas (TCGA-KIRC, https://cancergenome.nih.gov/) database. A total of 507 patients with follow-up information were identified and used for further analysis. The total RNA expression data were standardized through log2 transformation.

Seven pairs of the matched frozen samples of ccRCC and benign renal tissue adjacent to cancer used in this study were all from the Guangzhou First People’s Hospital. The included patients did not receive chemotherapy or radiotherapy before surgery. Each case was diagnosed and graded by two pathologists separately and re-examined by hematoxylin-eosin staining.

### Construction of a prognostic TIL-related lncRNAs signature in ccRCC

The correlation between lncRNAs and TIL-related genes (18) was calculated using the Pearson correlation analysis. The correlation coefficient |R| > 0.3 and *P*< 0.001 were the criteria for TIL-related lncRNAs. Univariate Cox regression was applied to assess the prognostic value of TIL-related lncRNAs. TIL-related lncRNAs with *P*< 0.001 in univariate analysis were incorporated into least absolute shrinkage and selection operator (Lasso) regression. Then, TIL-related lncRNAs identified by Lasso were included in a multivariate Cox model to establish a risk score. Finally, we identified 10 TIL-lncRNAs associated with the prognostic risk to construct a prognostic risk score. The risk score of KIRC patients was calculated as follows: Risk score = $\sum_{i=1}^{n}\beta_{i}\times \left(expression\;of\;lncRNA_{i}\right)$ . Patients in the cohort were divided into high- and low-risk groups according to the median risk score.

### Validation of the TIL-related lncrnas signature in ccRCC

Based on the TCGA-KIRC cohort, the expression of TIL-related lncRNAs was measured by ‘tinyarray’ R package. The Kaplan–Meier (K–M) survival curves of overall survival (OS) were used to evaluate the clinical prognostic value of TIL-related lncRNAs. Cytoscape software 3.7.2 was applied to visualize coexpression networks between TIL-related lncRNAs and TIL-related genes. Sankey diagram was used to assess the association between prognostic TIL-related lncRNAs, TIL-related genes, and risk types. The K–M survival curves were created to compare the overall survival of high- and low-risk groups according to predictive signatures. The receiver operating characteristic (ROC) curve from the “survivalROC” R package was used to assess the sensitivity and specificity of the signature. Univariate Cox regression and multivariate Cox regression were applied to assess the prognostic value of the signature.

### Construction and validation of a nomogram

Based on age, clinical stage, risk score, and pathological grade, a nomogram for OS was developed with the R package “rms” to predict the 1-year, 3-year, and 5-year relapse-free survival probability. The concordance index (C-index) was used to evaluate the consistency between the nomogram-predicted results and the actual observed results. A calibration curve was used to show the difference between the predictions of the model and the real outcomes. ROC analysis was performed to evaluate the predictive ability of the risk score. The web-based OS probability calculators were built using packages “DynNom” and “shiny” in R software.

### Functional enrichment

Differentially expressed genes (DEGs) of mRNA between the high-risk and low-risk groups were identified using package limma in R, with thresholds of |log2 fold change (FC)| > 2 and adjusted *P*< 0.05. Then, gene ontology (GO) enrichment analysis was performed to find the major biological process (BP) related to immune DEGs. The visual GO enrichment maps of the annotation analysis results were obtained by R with the “ggplot2” and “GOplot” packages.

### Somatic mutation analysis

Mutation data of the high-risk group and the low-risk group were analyzed and visualized using the “maftools” package. Mutation information for each gene in each sample was demonstrated by waterfall plots.

### Landscape of tumor-infiltrating immune cells based on the signature

To evaluate the effect of the signature on immune cells, CIBERSORT (Cell-type Identification by Estimating Relative Subsets of RNA Transcripts; http://cibersort.stanford.edu) was used to measure the abundance of tumor-infiltrating immune cells in a gene expression matrix by linear support vector regression (19). The abundance of lymphocyte profiles in the TCGA-KIRC dataset was obtained *via* the “CIBERSORT” R package. The infiltration levels in the high-risk group and the low-risk group were visualized by the “ggplot” R package.

### Real-time quantitative PCR assay

Total RNA was extracted from ccRCC and corresponding benign renal tissue samples using NucleoZOL reagent (740404.200, Meilunbio). Total RNA was then reverse-transcribed into cDNA using HiScript^®^ III RT SuperMix for qPCR (R323-01, Vazyme, Nanjing, China). Real-time quantitative PCR (RT-qPCR) was carried out using the AceQ^®^ qPCR SYBR Green Master Mix (Q141-02, Vazyme). The relative expression of lncRNA was calculated based on the internal reference β-actin. All experiments were carried out in three replicates. The primers applied are listed in **Supplementary Table S1**.

### Western blot assay

Quantitative analysis of protein expression in clinical tissues was performed using western blotting in accordance with the protocol of our previous study (20). The following antibodies were applied in the assay: β-Actin (1:5000; ab8227, Abcam, Cambridge, UK), CD4 (1:2000; 19068-1-AP, Proteintech, Chicago, IL, USA), CD8α (1:2000; 66868-1-Ig, Proteintech), PD-1 (1:2000; 66220-1-Ig, Proteintech), FoxP3 (1:2000; 22228-1-AP, Proteintech), and PD-L1 (1:2000, PTM-5075, PTM BIO, Hangzhou, China). Correlations between the expression levels of lncRNAs and immune markers were determined by Pearson correlation analysis.

### Transfection of cell lines

Human AC084876.1-specific siRNA and negative control siRNA were purchased from Tsingke Biotechnology Co., Ltd. (Beijing, China). The siRNA sequences are shown in the **Supplementary Table S1**. RT-qPCR analysis was conducted 72 h after transfection to test the transfection efficacy.

### Cell proliferation assays

Cell proliferation was measured by CCK-8 (Cell Counting Kit-8, MA0218, Meilunbio) assay as previously described (20).

### Cell migration and invasion assay

Cell migration and invasion were measured by a wound healing assay and transwell assay in line with the protocol from our previous study (20).

### Quantification of transforming growth factor-beta

The assay was conducted using a human TGF-β ELISA kit (JM-04929H1, Jingmei, Jiangsu, China). The cell culture medium was tested in accordance with the manufacturer’s instructions. The results were normalized using cell counts.

### Statistical analysis

All statistical analyses were performed in R software (version 4.03) and GraphPad Prism 8 (GraphPad Software, United States). Continuous variables are shown as the means ± standard deviations. Student’s *t* test or analysis of variance (ANOVA) was used to determine the statistical significance of quantitative data. *P*< 0.05 was regarded as statistically significant.

## Results

### Identifying 10 TIL-related lncRNAs in 507 patients with ccRCC

**Figure 1:**
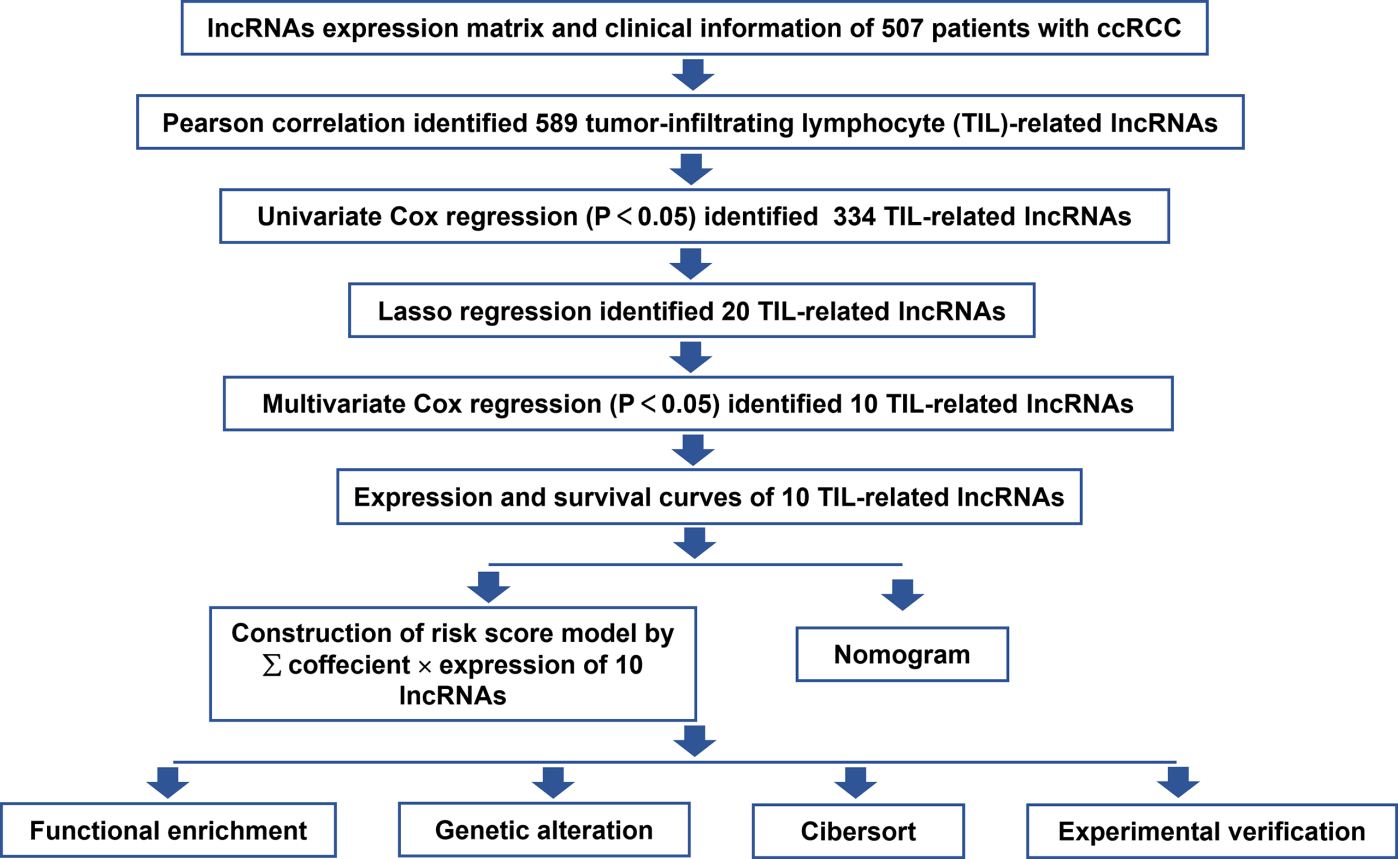
Flowchart of the study strategy.

**Figure 2:**
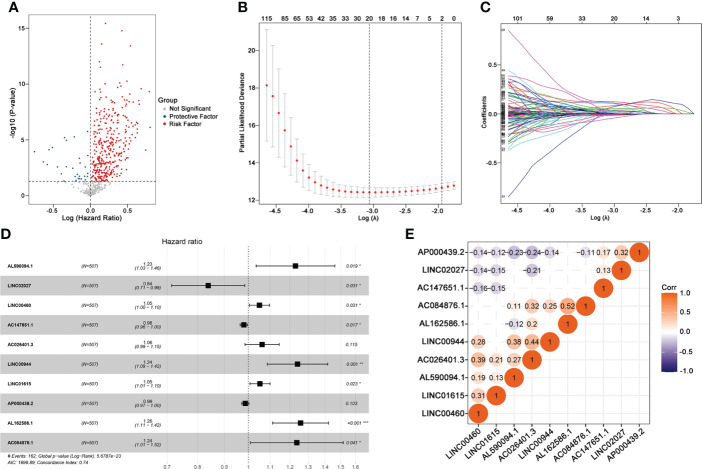
Identifying 10 TIL-related Long noncoding RNAs (lncRNAs)s. **(A)** Prognostic factors identified by univariate Cox regression. **(B)** Cross-validation for tuning parameter selection in the proportional hazards model. **(C)** Profiles of Lasso coefficients. **(D)** Multivariate Cox model of 10 TIL-related lncRNAs. **(E)** Correlation analysis between 10 TIL-related lncRNAs. “Corr” indicates the correlation coefficient generated by Pearson’s correlation analysis. *P < 0.05; **P < 0.01; ***P < 0.001.

This study was performed following the workflow shown in **Figure 1**. A total of 589 candidate lncRNAs related to tumor-infiltrating lymphocytes (TIL) were identified by Pearson’s correlation analysis (**Supplementary Table S2**). To further evaluate the prognostic value of these candidate lncRNAs, we performed univariate Cox regression analysis with the p-value of 0.05 as the cutoff threshold; 334 lncRNAs were detected as prognostic TIL-lncRNAs (**Figure 2A** and **Supplementary Table S3**). Next, by leveraging the Lasso algorithm, these 334 TIL-lncRNAs were narrowed down to 20 with the optimal lambda of 0.06175523 (**Figures 2B, C** and **Supplementary Table S4**). Furthermore, among the 20 TIL-lncRNAs, 10 lncRNAs were found by multivariate Cox regression analysis to be independent prognostic factors in ccRCC (**Figure 2D** and **Supplementary Table S5**). Among these 10 TIL-lncRNAs, seven lncRNAs, i.e., AL590094.1 (HR = 1.23, 95% CI 1.03 to 1.16, *P* = 0.019), LINC00460 (HR = 1.05, 95% CI 1.00 to 1.10, *P* = 0.031), AC026401.3 (HR = 1.06, 95% CI 0.99 to 1.15, *P* = 0.115), LINC00944 (HR = 1.24, 95% CI 1.09 to 1.42, *P* = 0.001), LINC01615 (HR = 1.05, 95% CI 1.01 to 1.10, *P* = 0.023), AL162586.1 (HR = 1.26, 95% CI 1.11 to 1.42, *P*< 0.001), and AC084876.1 (HR = 1.24, 95% CI 1.01 to 1.52, *P* = 0.041) served as negative prognostic factors, while other three lncRNAs, i.e., LINC02027 (HR = 0.84, 95% CI 0.71 to 0.98, *P* = 0.031), AC147651.1 (HR = 0.98, 95% CI 0.96 to 1.00, *P* = 0.017), and AP000439.2 (HR = 0.99, 95% CI 0.97 to 1.00, *P* = 0.103) were favorable prognostic factors in ccRCC.

Next, correlation analysis was conducted. As shown in **Figure 2E**, AC084876.1, LINC00944, and AC026401.3 had an obvious positive correlation with other lncRNAs, whereas AP000439.2 had a negative correlation with other lncRNAs. Taken together, 10 TIL-lncRNAs, which significantly correlated with TIL and were significant in the univariate Cox regression model, LASSO algorithm, and multivariate Cox proportional hazards regression model, were selected as the most important TIL-lncRNAs for further analysis.

### Construction of a TIL-related lncRNA prognostic signature

**Figure 3:**
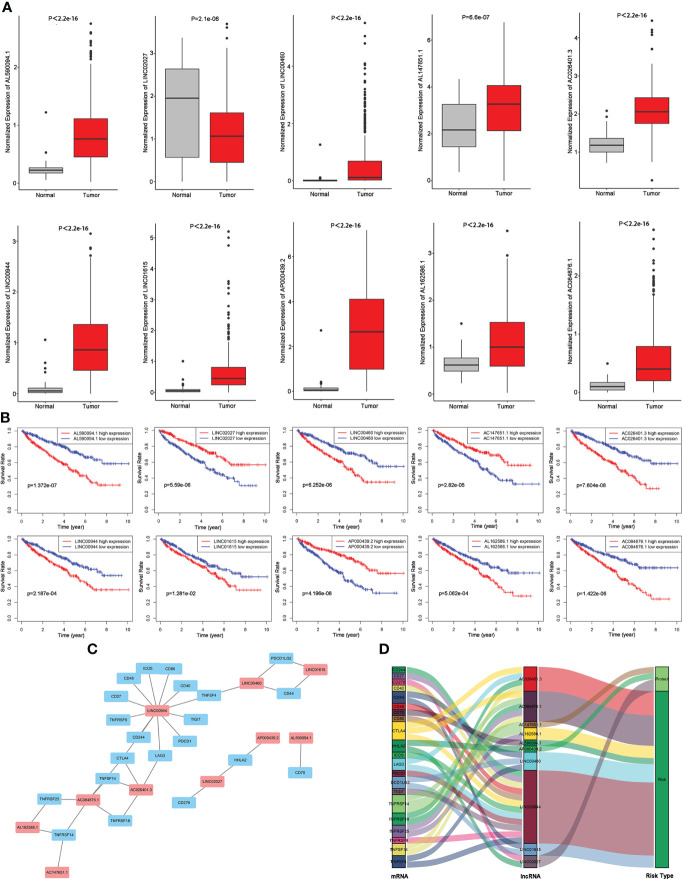
Evaluation of the 10 TIL-related lncRNAs. **(A)** Expression of the 10 TIL-related lncRNAs. **(B)** Prognostic value of the 10 TIL-related lncRNAs. **(C)** Coexpression network between prognostic lncRNAs and TIL-related genes. **(D)** Sankey diagram showing the association between prognostic TIL-related lncRNAs, TIL-related genes, and risk types.

We compared the abundance of 10 candidate TIL-lncRNAs between tumor and normal tissue in TCGA-KIRC by Wilcoxon signed-rank test and found that AL590094.1 (*P*< 2.2e-16), LINC00460 (*P* = 2.1e-06), AC147651.1 (*P* = 6.6e-07), AC026401.3 (*P*< 2.2e-16), LINC00944 (*P*< 2.2e-16), LINC01615 (*P*< 2.2e-16), AP000439.2 (*P*< 2.2e-16), AL162586.1 (*P*< 2.2e-16), and AC084876.1 (*P*< 2.2e-16) were significantly upregulated, while LINC02027 (*P* = 2.1e-06) was significantly downregulated in tumor tissue compared with normal tissue (**Figure 3A**). The prognostic value of the 10 TIL-lncRNAs was determined by K–M survival analysis (**Figure 3B**). Based on the median expression, patients were classified into high- and low-expression groups. Patients with high expression of AL590094.1, LINC00460, AC026401.3, LINC00944, LINC01615, AL162586.1, and AC084876.1 had poorer overall survival than those with low expression, whereas patients with high expression of LINC02027, AC147651.1, and AP000439.2 had better overall survival than those with low expression (log-rank test, all *P*< 0.05, **Figure 3B**), indicating that these 10 TIL-lncRNAs play a role in ccRCC. Considering that lncRNAs may participate in the splicing, maturation, transportation or localization, and stability of messenger RNAs (mRNAs), and thus regulate the translation and biological functions of mRNA (21), we explored the relationship between TIL-lncRNAs and TIL-mRNAs by conducting coexpression networks (**Figure 3C**) and summarized their relationships with risk type in Sankey diagram (**Figure 3D**). Briefly, AC026401.3, AP000439.2, and LINC02027 were protective factors related to TNFRSF14, HHLA2, and CD276. The other seven lncRNAs were risk factors related to CTLA4, LAG3, TNFRSF18, TNFSF14, TNFRSF14, TNFRSF25, CD70, CD44, DCD1LG2, TNFSF4, CD244, CD27, CD40, CD48, CD86, CTLA4, ICOS, LAG3, PDCD1, TIGIT, and TNFRSF9.

Then, 10 TIL-lncRNAs were enrolled to construct a TIL-related lncRNA signature with the following formula for the risk score: Risk score = (0.2068 × expression level of AL590094.1) + (−0.1762 × expression level of LINC02027) + (0.0487 × expression level of LINC00460) + (−0.0199 × expression level of AC147651.1) + (0.0604 × expression level of AC026401.3) + (0.2158 × expression level of LINC00944) + (0.0509 × expression level of LINC01615) + (−0.0134 × expression level of AP000439.2) + (0.2292 × expression level of AL162586.1) + (0.2125 × expression level of AC084876.1).

### Evaluation of the 10 TIL-lncRNAs signature

**Figure 4:**
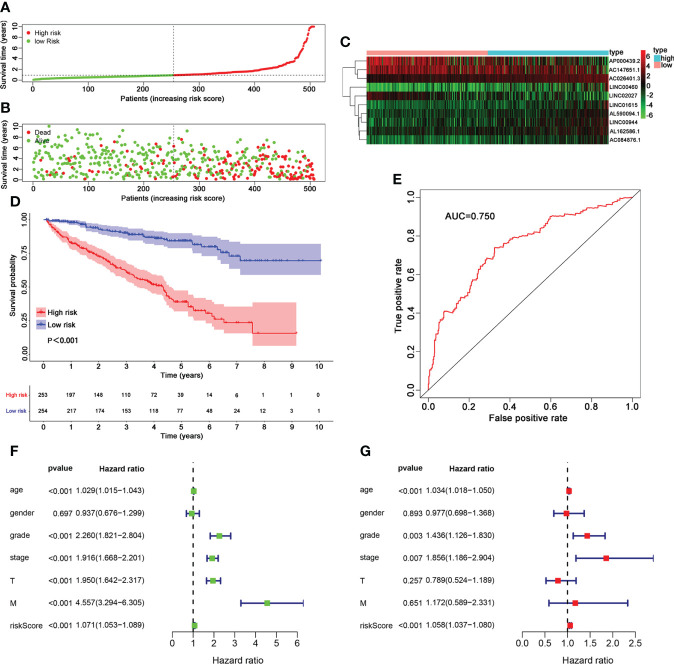
Analysis of TIL-related lncRNA signature for patients with ccRCC. **(A–C)** lncRNA predictor-score analysis of 507 ccRCC patients. The horizontal axis represents the 507 patients. Heat map of lncRNA expression level. **(D)** Survival time of patients between the groups. **(E)** Areas under the ROC curve. Forest plots for univariate **(F)** and multivariate **(G)** Cox regression analysis.

Based on the median cutoff value, patients in the cohort were divided into the high-risk group (n = 253) and the low-risk group (n = 254) (**Figure 4A**). As shown in **Figure 4B**, the death probability of high-risk patients was higher than that of low-risk patients. It was also found that the expression levels of AC026401.3, LINC00944, and AC084876.1 were visibly higher, and the expression levels of AP00439.2, AC14765.1, and LINC02027 were downregulated in the high-risk group (**Figure 4C**).

The K–M survival curves showed that patients with a high risk score had significantly poorer overall survival than those with a low risk score (**Figure 4D**). Then, time-dependent ROC curves were used to assess the predictive performance of the TIL-related lncRNA signature (**Figure 4E**). The area under the ROC (AUC) was 0.750, indicating that the TIL-lncRNA signature was a reliable prognostic indicator for predicting OS in ccRCC. In addition, univariate Cox regression and multivariate Cox regression analysis were employed to assess the independent prognostic value of the signature with the following factors: risk score and relevant clinical factors (age, gender, grade, clinical stage, tumor stage [T], and metastasis stage [M]). Node stage was excluded as much data were missing. All of the factors except gender were significantly associated with OS in univariate analysis (**Figure 4F**). Multivariate analysis indicated that risk score was still significantly related to OS, suggesting the TIL-related lncRNA signature could serve as an independent prognostic factor for patients with ccRCC (**Figure 4G**).

### Construction of a TIL-related lncRNA prognostic model to predict the survival

**Figure 5:**
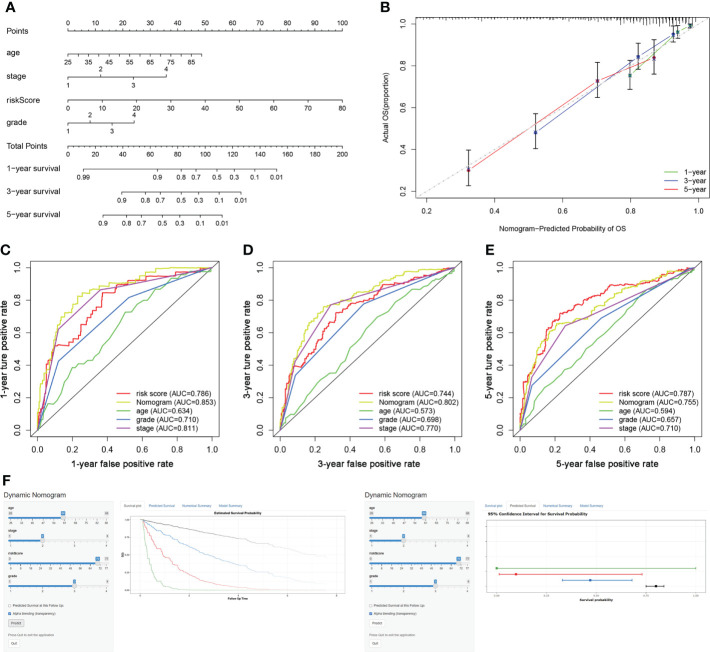
Construction of a TIL-related lncRNA prognostic model to predict survival. **(A)** The nomogram of 1-year, 3-year, or 5-year OS. **(B)** Calibration plots for evaluating the agreement between the predicted and the actual 1-year, 3-year, and 5-year OS for the prognosis model. **(C–E)** The 1-year, 3-year, and 5-year ROC curves of the nomogram and other clinicopathological parameters. **(F)** A dynamic nomogram for clinical application to predict survival. The K–M analysis is shown on the left. The corresponding 95% CI is shown on the right.

The risk score, age, clinical stage, and pathologic grade were included in the nomogram. As indicated in the nomogram, the risk score had the largest contribution to OS of patients with ccRCC (**Figure 5A**). The C-index of the nomogram was 0.693. The calibration curve revealed good agreement between the predicted and observed probabilities. All calibration curves of 1-year, 3-year, and 5-year (**Figure 5B**) OS were close to the 45-degree line.

As shown in **Figures 5C–E**, the area under curve (AUC) of the nomograms was 0.853, 0.802, and 0.755 for 1-, 3-, and 5-year OS, respectively. These AUC values of the nomograms were greater than those of every single clinical predictor (i.e., age, grade, and stage), indicating an advantage of combining these risk factors for ccRCC prognosis.

For the clinical usability of the model, a dynamic nomogram was created for the prediction of OS probability in patients with ccRCC (**Figure 5F**), which was convenient and intuitive for individual prognosis prediction based on the personal characteristics of ccRCC patients (https://zhonglab.shinyapps.io/dynnomapp/).

### Functional analysis

**Figure 6:**
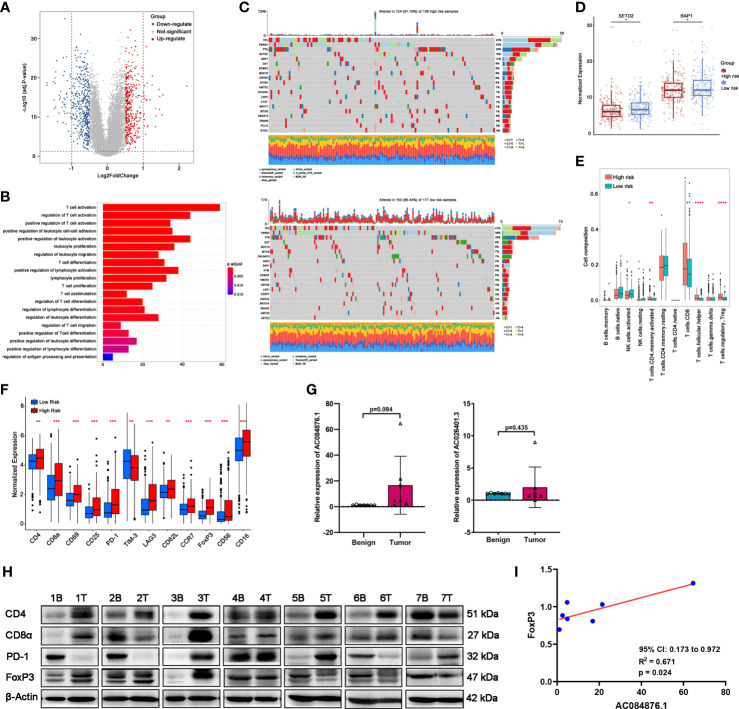
Correlation between the signature and TILs in ccRCC tissues. **(A)** DEGs between the groups by volcano map. **(B)** GO-BP enrichment analysis. **(C)** Waterfall plots represent mutation information in each ccRCC patient sample in the high- and low-risk groups. **(D)** Expression of SETD2 and BAP1. **(E)** TILs composition analysis in ccRCC of TCGA cohort. **(F)** Expression of immune markers of lymphocytes of TCGA cohort. **(G, H)** The expression of AC084876.1 and AC026401.3 in seven kidney samples from ccRCC patients. **(H)** The expression of CD4, CD8α, PD-1, and FoxP3 in seven kidney samples from ccRCC patients. “B” indicates benign renal tissue adjacent to cancer. “T” indicates tumor tissues of ccRCC. **(I)** Correlation between the expression of AC084876.1 and FoxP3 in seven ccRCC samples. *P < 0.05; **P < 0.01; ***P < 0.001; ****P < 0.0001.

As shown in the Volcano plots (**Figure 6A**), DEGs were identified with fdr< 0.05 and |logFC| > 0.5. A total of 408 genes were upregulated and 455 genes were downregulated in the low-risk group compared with the high-risk group (**Supplementary Table S6**). GO enrichment analysis (**Figure 6B**) indicated that these DEGs were mostly related to the pathways involved in T-cell activation, differentiation, proliferation, co-stimulation, and migration.

### Prediction of the immune infiltration

Somatic mutations often affect the distribution of tumor-infiltrating immune cells in the tumor microenvironment, thereby causing tumor heterogeneity. The results of somatic mutation analysis (**Figure 6C**) indicated that the top five mutated genes in the high-risk group were VHL (43%), PBRM1 (40%), TTN (26%), SETD2 (18%), and BAP1 (14%). The top five mutated genes in the high-risk group were VHL (41%), PBRM1 (38%), TTN (26%), DST (8%), and MUC16 (8%). Considering the higher mutation frequency of SETD2 and BAP1 in the high-risk group, we measured their expression levels. The expression levels of SETD2 and BAP1 were downregulated in the high-risk group (**Figure 6D**).

CIBERSORT analysis (**Figure 6E**) indicated that the abundance of activated CD4+ memory T cells, CD8+ T cells, CD4+ follicular helper T cells (TFH), and regulatory T cells (Tregs) was higher in the high-risk group. A higher abundance of activated NK cells was observed in the low-risk group. As shown in **Figure 6F**, immune markers CD4, CD8α, CD69, CD25, PD-1, LAG3, CD62L, CCR7, FoxP3, CD56, and CD16 were significantly upregulated in the high-risk group with a decrease in TIM-3.

### Measuring the expression levels of AC084876.1 and AC026401.3 and immune markers in kidney tissues

Considering the above results, we performed an RT-qPCR assay to detect the expression of AC084876.1 and AC026401.3 in seven pairs of matched frozen samples of ccRCC and benign renal tissue adjacent to cancer from ccRCC patients. Moreover, a western blot assay was conducted to measure the expression of immune markers (including CD4, CD8α, PD-1, and FoxP3, **Figure 6H**). As shown in **Figure 6G**, the expression of AC084876.1 and AC026401.3 tended to be higher in cancer tissues although the threshold of statistical significance was not reached. The results of the western blot showed a higher trend for all of the immune markers detected in cancer tissues, but without reaching statistical significance (**Figure S1A**). For the correlation analysis, we found that the expression of AC084876.1 positively correlated with FoxP3 (**Figure 6I**, 95% CI 0.173 to 0.972, R^2^ = 0.671, *P* = 0.024). No statistically significant results were found in other correlation analyses (**Figure S1B**).

### Selecting AC084876.1 for experimental validation

**Figure 7:**
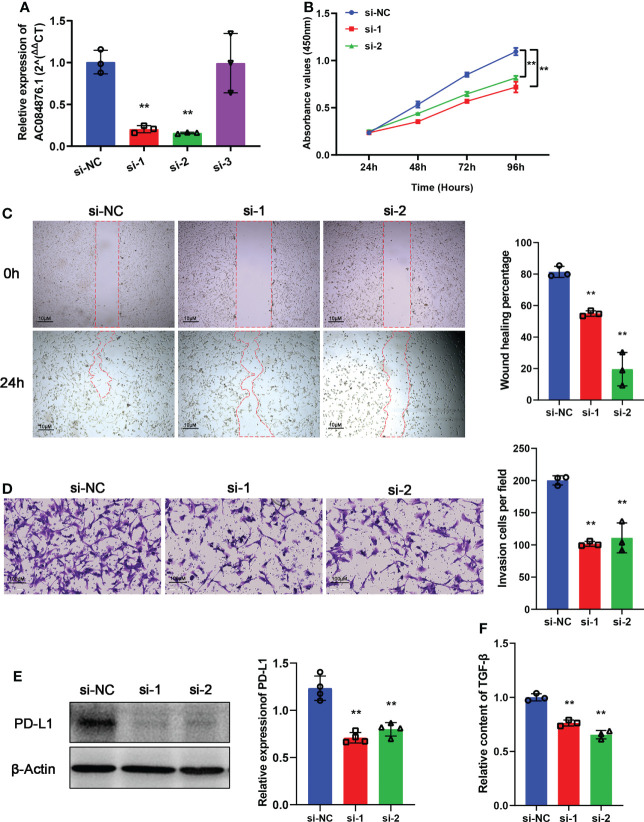
Experimental validation of AC084876.1. **(A)** Transfection efficiency of siRNAs targeting AC084876.1 in the 786-O cell line. **(B)** Growth curve of AC084876.1-downregulated 786-O cell line. **(C, D)** Knockdown of AC084876.1 inhibited migration and invasion of the 786-O cell line. **(E)** Eexpression of PD-L1 decreased in the AC084876.1-downregulated 786-O cell line. **(F)** TGF-β secretion decreased in the AC084876.1-downregulated 786-O cell line. **P < 0.01.

siRNAs were used to knock down the expression of AC084876.1 in the 786-O cell line. The transfection efficiency was measured by RT-qPCR, and we showed that si-1 and si-2 significantly interfered with the expression of AC084876.1 (**Figure 7A**, *P*< 0.01). The growth curve suggested that the knockdown of AC084876.1 inhibited the growth of the 786-O cell line (**Figure 7B**, *P*< 0.01). The migration ability (**Figure 7C**, *P*< 0.01) and invasion ability (**Figure 7D**, *P*< 0.01) were attenuated in AC084876.1-downregulated 786-O cell lines.

PD-L1 expressed on the surface of tumor cells and TGF-β secreted by tumor cells have been shown to regulate the activity and differentiation of tumor-infiltrating Tregs (22). To further analyze the potential role of AC084876.1 to regulate tumor-infiltrating Tregs, the expression level of PD-L1 and secreted TGF-β level were also examined. We found that the knockdown of AC084876.1 resulted in decreased expression of PD-L1 (**Figure 7E**, *P*< 0.01) and decreased secreted TGF-β (**Figure 7F**, *P*< 0.01).

## Discussion

ccRCC is the most common pathological subtype of renal carcinoma. Although at an early stage patients can benefit from surgical treatment, advanced patients have finite treatment options (23). Immunotherapy has ignited their hope, but the treatment effectiveness is still limited. The important role of lncRNAs in tumor progression has been gradually explored. To date, several prognostic models based on the immune-related lncRNAs have suggested that lncRNAs are involved in the regulation of immune cell–mediated tumor killing in the tumor microenvironment (TME) (24–26). A large number of immune cells infiltrate the TME, and tumor-infiltrating lymphocytes (TIL) play a key role in the response to immunotherapy (27). Thus, it is worth noting that investigating the potential role of TIL-related lncRNA may identify novel biomarkers for the treatment and prognosis of patients with ccRCC.

In the present study, we established a novel risk coefficient model based on TIL-related lncRNAs through Pearson correlation analysis, univariate Cox regression, Lasso regression, and multivariate Cox regression in 507 ccRCC patients from the TCGA-KIRC dataset. Finally, 10 TIL-related lncRNAs (AL590094.1, LINC02027, LINC00460, AC147651.1, AC026401.3, LINC00944, LINC01615, AP000439.2, AL162586.1, and AC084876.1) were identified and included to construct the prognostic signature. Considering the results of survival analysis, Sankey diagram, and expression analysis of lncRNAs in the high-risk group, AC026401.3, LINC00944, and AC084876.1 were risk factors, whereas AP00439.2, AC14765.1, and LINC02027 were protective factors. Exploring the expression of biomarkers in experiments, we found that the expression trends of AC084876.1 and AC026401.3 in benign and malignant kidney tissues were consistent with the results of bioinformatics analysis, although the differences did not reach the threshold of statistical significance. A larger sample size is needed for further confirmation. The overall survival of ccRCC patients in the TCGA cohort with high expression of AC084876.1 was worse, and AC084876.1 was present as a risk factor in the prognostic model. The experimental validation indicated that the knockdown of AC084876.1 inhibited the growth, migration, and invasion ability of the ccRCC cell line *in vitro*. Thus, lncRNAs in the signature were tightly related to TILs and reliable to further investigate the potential role of TIL-related lncRNA signature in the progression of ccRCC.

The survival analysis indicated that the survival rate of patients in the low-risk group was significantly higher. Univariate Cox regression and multivariate Cox regression analysis showed that the risk score could be an independent predictor of overall survival in patients with ccRCC. In the nomogram model, risk score contributed the most, and the calibration curves of 1-, 3-, and 5-year survival prediction were close to the ideal value. Also, the AUC values of the 1-, 3-, and 5-year nomograms were greater than those of every single clinical predictor, indicating that the nomogram could be clinically helpful. To make the model applicable for clinical use, we constructed a dynamic nomogram for convenient and intuitive individual prognosis prediction based on the personal characteristics of ccRCC patients.

Consistent with previous studies based on the immunotherapy effectiveness in ccRCC (28, 29), we found that the mutation frequencies of SETD2 and BAP1 were higher and their expression were both downregulated in the high-risk group. SETD2 and BAP1 mutations are associated with metastasis and poor prognosis in ccRCC (30, 31). SETD2 mutations also play an important role in promoting ccRCC progression through cellular autophagy inhibition, DNA repair inhibition, and genomic stability perturbation (32, 33). Mutation of BAP1 may engender genomic instability and promote defects in DNA repair pathways (34). Thus, our novel TIL-related lncRNA signature may contribute to the understanding of the potential mechanism of the progression of ccRCC.

GO-BP analysis of DEGs showed that the immune-related biological processes were mostly involved in T-cell activation, differentiation, proliferation, co-stimulation, and migration. By applying CIBERSORT, we found that the abundance of activated CD4+ memory T cells, CD8+ T cells, TFH, and Tregs was higher in the high-risk group. CD8+ T cells are the main executors of killing tumor cells in the immune system. However, high abundance of CD8+ T cells was not associated with a favorable prognosis in ccRCC (35), and it has been reported that CD8+ T cells did not show functional status as exhausted CD8+ TILs (36). Consistently, our study showed that the exhausted T-cell markers, including PD-1, LAG3, and FoxP3, were upregulated in the high-risk group. Tumor-infiltrating CD8+T cells lose the ability to recognize antigens and activate proliferation under the long-term effect of inhibitory cells and factors, thereby leading to the failure of tumor-killing function (37). Moreover, as an important suppressive immune cell, Tregs can inhibit the proliferation of CD8+T cells by secreting TGF-β, IL-10, and IL-35 (38).

Our results demonstrated that the expression of AC084876.1 positively correlated with FoxP3 in ccRCC. FoxP3 is considered an important biomarker to characterize the Tregs, which are an immunosuppressive subset of CD4+ T cells (39, 40). It is widely accepted that Tregs, the central mediators of immune suppression, are activated by PD-L1 and are induced to differentiate by TGF-β synthesized from tumor cells (22). PD-L1 expressed on tumor cells interacts with PD-1 on tumor-infiltrating lymphocytes, attenuating effector T-cell responses and allowing tumors to escape immune attack (41). In addition, Foxp3 in naive T cells could be induced by TGF-β, thereby promoting Tregs development (42). Interestingly, we found that knockdown of AC084876.1 resulted in decreased expression of PD-L1 and reduced level of secreted TGF-β. These results suggest that AC084876.1 may play an important role in the progression of ccRCC by regulating the activation and differentiation of Tregs. Tumor-infiltrating Tregs are highly activated (43) and exert suppressive activities on effector cells by inducing apoptosis and inhibiting activation/proliferation (44). It has been reported that Tregs promote tumorigenesis and immunosuppression *via* increasing consumption of IL-2 and upregulating inhibitory immune checkpoints (45). Importantly, the functional enrichment analysis suggested that our signature was related to T-cell activation, differentiation, and proliferation signaling pathways, and Tregs were significantly increased in the high-risk groups. Thus, we believe this may be related to the potential regulatory mechanism between AC084876.1 and tumor-infiltrating Tregs, which is worthy of further investigation.

Despite a number of immune-related lncRNA signatures, to our knowledge, the present study was the first to identify a TIL-related lncRNA signature to predict the prognosis of ccRCC patients. Compared with previous studies, which only established signatures (26, 46), our novel signature was used to establish a nomogram combined with clinical indicators to accurately predict survival. As for the study with nomogram construction (24), the potential mechanism of our signature was verified with experiments. We showed that the signature was mainly related to the function of T cells. Furthermore, we found that AC084876.1 may serve as a potential therapy target associated with the activation and differentiation of tumor-infiltrating Tregs. However, our study was retrospective, and a larger validation cohort is needed to confirm our conclusions. Moreover, the underlying mechanism of the identified lncRNAs in regulating TILs and tumor progression in ccRCC remains to be further explored.

## Data availability statement

The original contributions presented in the study are included in the article/**Supplementary Material**. Further inquiries can be directed to the corresponding authors.

## Ethics statement

The studies involving human participants were reviewed and approved by the Ethics Committee of Guangzhou First People’s Hospital, School of Medicine, South China University of Technology. The patients/participants provided their written informed consent to participate in this study.

## Author contributions

WZ, GZ, and ZH supervised the whole project and participated in study design and coordination. YD and KG analyzed data and wrote and revised the paper. YD, ZFT, and YF processed and performed the bioinformatic analysis. SC, YX, JL, RL, ZHT, and YZ collected clinical tissues and performed the experimental assays. YF, JY, and CC checked the revised the figures. All authors contributed to the article and approved the submitted version.

## Funding

This work was supported by grants from the National Natural Science Foundation of China 82072813 (WZ); Guangdong Basic and Applied Basic Research Foundation 2017A030310100 (KG); Science and Technology Projects in Guangzhou 202201010726 (ZH); Science and Technology Development Fund of Macau SAR 0031/2021/A (WZ); Emergency Key Program of Guangzhou Laboratory EKPG21-04 (WZ); Guangdong Basic and Applied Basic Research Foundation 2020A1515110792, 2022A1515010342 (JY).

## Conflict of interest

The authors declare that the research was conducted in the absence of any commercial or financial relationships that could be construed as a potential conflict of interest.

## Publisher’s note

All claims expressed in this article are solely those of the authors and do not necessarily represent those of their affiliated organizations, or those of the publisher, the editors and the reviewers. Any product that may be evaluated in this article, or claim that may be made by its manufacturer, is not guaranteed or endorsed by the publisher.
