# Supplementary material for: Identification and experimental validation of a tumor-infiltrating lymphocytes–related long noncoding RNA signature for prognosis of clear cell renal cell carcinoma
Source: Front Immunol. 2022 Nov 24;13:1046790. doi: 10.3389/fimmu.2022.1046790 (PMC9730408; doi:10.3389/fimmu.2022.1046790)
Supplement: Supplementary file 1 [file Table_1.docx]

Table S1 Sequence of primers and siRNA applied in this study

| Primers |  | 5'-3' |
| --- | --- | --- |
| AC084876.1 | Forward | GCACCAATCTGTGAAAAATACACC |
|  | Reverse | TCTTTCACATTTAACAGTTGCTTGG |
| AC026401.3 | Forward | CCCACAGAGTGGTGAAAAACTT |
|  | Reverse | ACAAACACATCCCGGATACATT |
| β-Actin | Forward | AGCGAGCATCCCCCAAAGTT |
|  | Reverse | GGGCACGAAGGCTCATCATT |

| siRNA | | |
| --- | --- | --- |
| AC084876.1 |  | 5'-3' |
| si-1 | sense | GGCUGGUCUUGAACUCCUA |
|  | antisense | UAGGAGUUCAAGACCAGCC |
| si-2 | sense | GAGACUUAUCCUACAAAUA |
|  | antisense | UAUUUGUAGGAUAAGUCUC |
| si-3 | sense | CUAUUAUCCUGAUGACAAA |
|  | antisense | UUUGUCAUCAGGAUAAUAG |
