# Supplementary material for: Identification and experimental validation of a tumor-infiltrating lymphocytes–related long noncoding RNA signature for prognosis of clear cell renal cell carcinoma
Source: Front Immunol. 2022 Nov 24;13:1046790. doi: 10.3389/fimmu.2022.1046790 (PMC9730408; doi:10.3389/fimmu.2022.1046790)
Supplement: Supplementary file 7 [file DataSheet_1.docx]

Figure s1. Expression of AC084876.1, AC026401.3 and immune markers in 7 kidney samples for ccRCC patients. (A) Quantification of the expression of CD4, CD8α, PD-1 and FoxP3 in 7 kidney samples from ccRCC patients. (B) Correlation between AC084876.1, AC026401.3 and immune markers.
