# Supplementary figures and images for: Identification and experimental validation of a tumor-infiltrating lymphocytes–related long noncoding RNA signature for prognosis of clear cell renal cell carcinoma
Source: Front Immunol. 2022 Nov 24;13:1046790. doi: 10.3389/fimmu.2022.1046790 (PMC9730408; doi:10.3389/fimmu.2022.1046790)

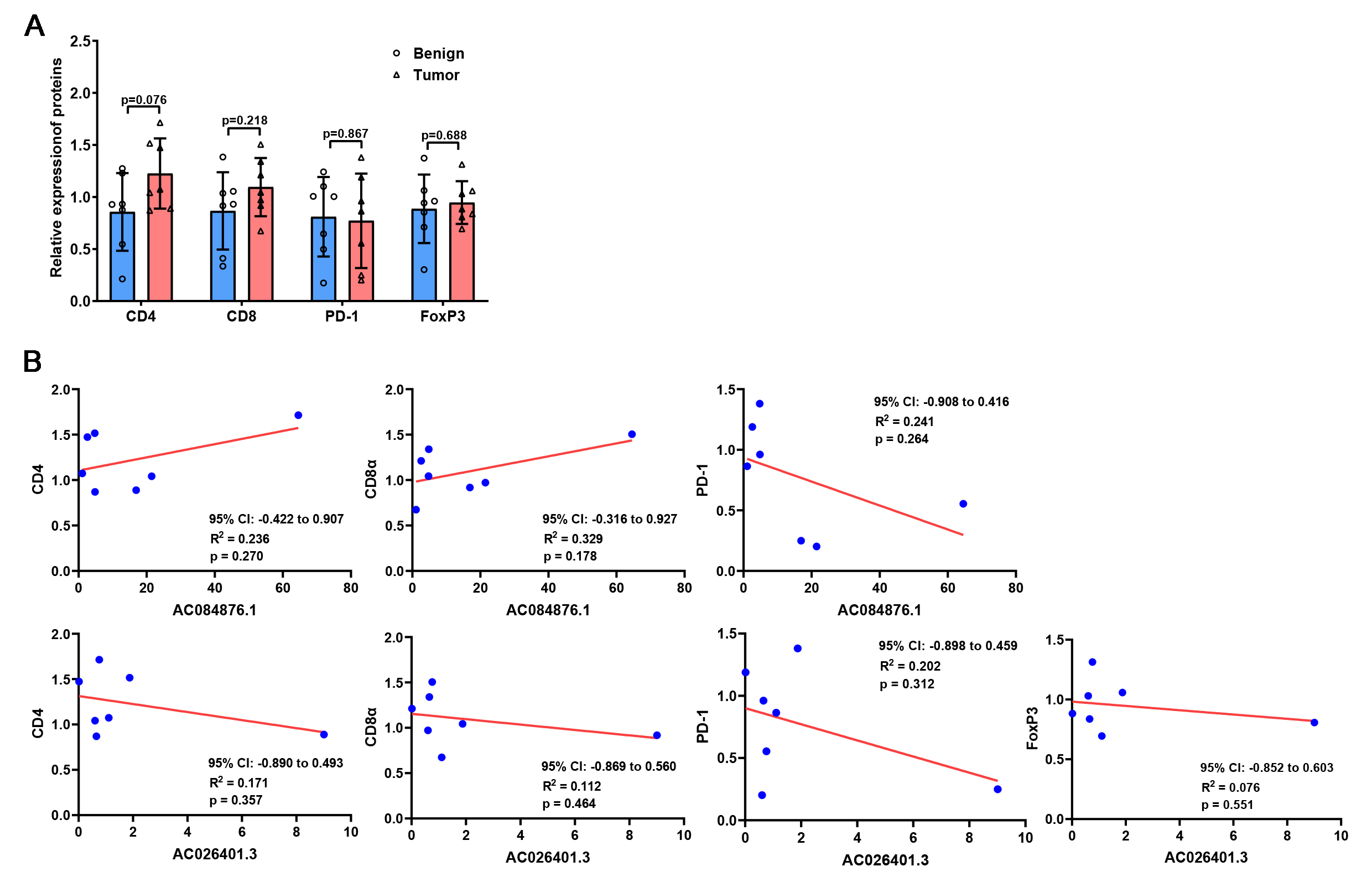

Supplement: Supplementary file 8 [file Image_1.tif]
